# Increasing incidence of premature thelarche in the Central Region of Denmark - Challenges in differentiating girls less than 7 years of age with premature thelarche from girls with precocious puberty in real-life practice

**DOI:** 10.1186/s13633-016-0022-x

**Published:** 2016-02-22

**Authors:** Mia Elbek Sømod, Esben Thyssen Vestergaard, Kurt Kristensen, Niels Holtum Birkebæk

**Affiliations:** Department of Pediatrics, Aarhus University Hospital, Skejby, Palle Juul Jensens Boulevard 99, DK-8200 Aarhus N, Denmark; Medical Research Laboratory, Aarhus University, Nørrebrogade 44 building 3B, DK-8000 Aarhus C, Denmark; Department of Pediatrics, Randers Regional Hospital, DK-8930 Randers, Denmark

**Keywords:** Sex hormone binding globulin, Premature thelarche, Incidence, Gonadotropins, Precocious puberty

## Abstract

**Background:**

Premature thelarche (PT) seems to be increasing and it is difficult to differentiate its early stages from precocious puberty (PP). Clinical and biochemical parameters are warranted to differentiate the two diagnoses.

**Methods:**

One hundred ninety-one girls aged 0.5–7 years were included. Diagnoses were validated and the girls were categorized to the groups PP (*n* = 27) and PT (*n* = 164). Anthropometry, Tanner stages, ethnicity, bone age, and biochemistry, were recorded. Conventional variables for diagnosing PP were compared between the groups at time of referral to identify parameters predictive for the diagnosis.

**Results:**

The referral rate of PT increased from 1998–2013. Girls with PT and PP differed with regards to age at referral, body mass index standard deviation scores (BMISDS), ethnicity, bone age advancement, basal luteinizing hormone (LH), gonadotropin releasing hormone (GnRH) stimulated LH and follicle stimulating hormone (FSH), basal and stimulated LH/FSH ratio, and sex-hormone binding globulin (SHBG). Apart from SHBG there was considerable overlap of the variables between the PT and the PP groups.

**Conclusions:**

First, the incidence of PT appears to increase. Second, SHBG was the variable which best discriminated PT from PP. Third, stimulated LH in 1–3 years old girls with PT is similar to stimulated LH in 5–7 years old girls with PP. Age, BMISDS, ethnicity, bone age, stimulated gonadotropins and LH/FSH and SHBG are all useful variables for differentiating PP from PT. However normative data for stimulated LH and FSH in the age group 0.5–7 years are warranted.

## Background

Onset of puberty before eight years of age for girls, so-called precocious puberty (PP), is increasing [1–3]. PP is associated with reduced adult height [4], psychosocial problems [5, 6], and may be associated with breast cancer [7] and the metabolic syndrome [8]. The first clinical sign of puberty in girls is usually breast development (thelarche). Thelarche is accompanied with accelerated growth velocity and bone age advancement. Early stages of PP in girls are, therefore, difficult to differentiate from premature thelarche (PT), which is defined as isolated breast development before eight years of age [9].

The frequency of PT seems dependent on ethnicity and may be increasing [10]. In an U.S. study the incidence rate of PT in the period 1940–1984 was 2.1 per 10,000 person years [11]. PT is a self-limiting condition in the majority of girls, but it may progress into PP in a subset of girls [12–14]. De Vries et al. and Pasquino et al. reported that 13 % and 14 %, respectively, of girls with PT progressed into PP [13, 14]. It is of great importance to identify the girls with PT, who progress into PP [9], and to initiate medical treatment to circumvent the negative implications of PP [15].

Unfortunately, robust clinical, biochemical and imaging indicators to help clinicians differentiate PT from PP are lacking, and no single test can predict the progression from PT to PP [12–14]. Diagnostic tests that may help to differentiate PT from PP include pelvic ultrasound measurements [16], bone age evaluation, basal luteinizing hormone (LH), and gonadotropin releasing hormone (GnRH) test, although normative data for the GnRH test in the first years of life have not yet been established.

The aim of this study was to test the hypothesis that the incidence of PT in girls in the Central Region of Denmark is increasing. A further aim was to describe challenges in differentiating girls with PT from girls with early PP using conventional variables for diagnosing PP in real-life practice.

## Methods

We identified girls aged 0.5–7 years referred for breast development between January 1998 and September 2013 to the pediatric departments in the Central Region of Denmark (population 1,277,538).

For screening purposes, all patient files of girls who were registered in the Danish National Patient Registry with the ICD10 codes as listed in Table 1, were carefully reviewed.

**Table 1:** ICD10-diagnosis codes used for the registry extraction

ICD10-code	Description
N60.X	Disorders of breast
N62.X	Hypertrophy of breast
N63.X	Unspecified lump in breast
N64.9	Disorder of breast, unspecified
E30.X	Disorders of puberty, not elsewhere classified
Q78.1	McCune Albright
E22.8	Central precocious puberty
E25.0	Congenital adrenal hyperplasia
E270B	Premature adrenarche
E25.X	Pseudopuberty

Codes where a diagnosis of premature thelarche or precocious puberty could potentially have been misclassified were included

The girls were included in the study, if they presented with uni- or bilateral breast development corresponding to Tanner stage 2 or more. Ninety-four girls did not meet the inclusion criteria and were subsequently excluded from further analysis. Girls were excluded because 1) they did not have breast development at the first visit (either because of regression or misdiagnosis or they had isolated adrenarche) and 2) data on breast development were missing in the patient file. Girls were assigned to the PP group if they, at the time of referral or before their seventh year, were diagnosed with PP by a pediatric endocrinologist. For validating girls into the PP group they were required to meet the following criteria: breast Tanner stage 2 or more combined with one or more of the following: pubic hair, accelerated growth velocity and bone age greater than 2 SD above the chronological age. Further, they should have a pubertal response (primarily assessed by the peak LH/FSH ratio and the LH response > 5 IU/L ) if they underwent a GnRH test at time of referral and data were available. For validating girls into the PT group they were required to have breast development corresponding to Tanner stage 2 or more, without any other signs of puberty.

The following parameters were extracted from the patient charts: Ethnicity, anthropometry, Tanner stages of breast and pubic hair, bone age [17], magnetic resonance imaging of the brain (MRI) and biochemistry: Estradiol, inhibin B, sex hormone-binding globulin (SHBG), thyroid stimulating hormone (TSH) luteinizing hormone (LH), follicle stimulating hormone (FSH), and the FSH and LH concentrations 30 min after an intravenous injection of 0.1 mg/m^2^ Relefact® (a GnRH agonist), hereafter designated stimulated LH and FSH concentrations.

Some turned 7 years before a GnRH test was performed. The presented biochemical data represent the results from the first blood samples after referral.

The number of newborn girls per year and girls in the age group 0–7 years in the Central Region of Denmark from 1998 to 2013, were obtained from ‘Statistics Denmark.’

The study was approved by the National Research Ethics Committee (reference number 1-10-72-186-13) and the Danish Data Protection Agency (reference number 1-16-02-118-13).

### Assays

Up to March 2008 LH, FSH, estradiol and TSH levels were measured by chemilu-minescence immunoassay (Siemens Bayer Advia Centaur CP Immunoassay). Since March 2008 LH, FSH, estradiol and TSH were measured by electrochemiluminescence immunoassay (Roche Cobas E 601, module immunology analyzer). SHBG levels were measured by chemiluminescence immunoassay (Siemens Bayer Advia Centaur CP Immunoassay) up to November 2010 and since then by electrochemiluminescence immunoassay (Roche Cobas E 601, module immunology analyzer). Serum levels of Inhibin B were measured by the Beckman Coulter GenII assay.

### Statistics

Statistical analysis was performed by SPSS software version 21. Linear distributed data are presented as mean and SD, while nonlinear distributed data are presented as median and range. Body mass index standard deviation score (BMI SDS) was calculated according to Nysom et al. [18]. An independent samples T test was used to compare the means of the parametric variables in the two groups (PP vs. PT). Some parameters were ln-transformed to obtain normal distribution. Non-parametric variables were compared using Mann-Whitney U test. Binary variables were analyzed using chi-square test. A difference was considered statistically significant at *p* < 0.05.

## Results

In total 285 patients were identified. Ninety-four patients did not meet the inclusion criteria and the remaining 191 girls (0.5 to 6.9 years) were included in the study and allocated to the PP group (*n* = 27) or the PT group (*n* = 164). One girl with PP was diagnosed with central PP (hamartoma of tuber cinereum), and the rest were diagnosed with idiopathic central PP. None of the girls was diagnosed with peripheral PP.

### Incidence

**Fig. 1:**
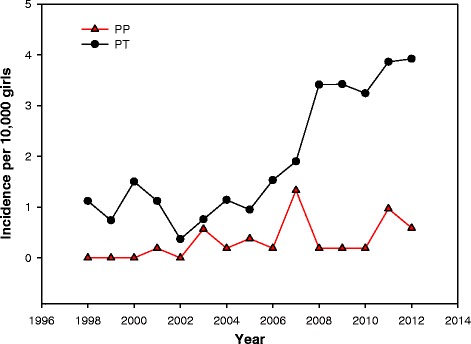
Incidence of precocious puberty and premature thelarche expressed as an incidence rate and defined as: $\frac{No.\  of\  girls\  who\ got\ a\  diagnosis\  of\ PP\  or\ PT\  in\ a\  certain\  year}{Total\  no.\  of\  girls\ 1/2-7\  year s\  living\  in\  the\  region}$

The annual incidence ranged from 0 to 1 and from 1 to 4 per 10,000 girls for PP and PT, respectively, (Fig. 1).

### Non-biochemical characteristics

**Fig. 2:**
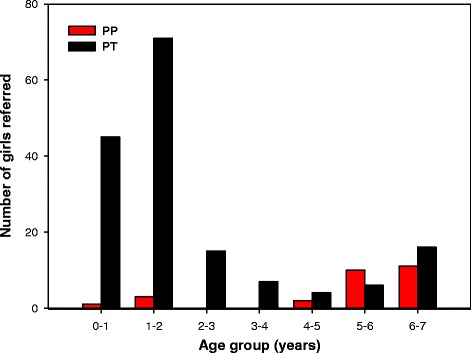
Number of girls with precocious puberty (PP) and premature thelarche (PT) in the age groups ½-7 years

Girls with PP were older at referral (*p* < 0.001) (Table 2): 14.8 % (*n* = 4) were in the age group 0.5–2 years, whereas 77.7 % (*n* = 21) were 5–7 years old. For girls with PT the majority (70.7 %, *n* = 116) was referred at age 0.5–2 years, Fig. 2.

**Table 2:** Clinical and biochemical data of girls with precocious puberty (PP) and premature thelarche (PT)

Variable	PP	n=	PT	n=	*P*-value
Age at referral (years)^a^	5.9 (1.0-6.9)	27	1.3 (0.5-6.9)	164	0.000
European origin (%)	70.4	27	89.6	164	0.012
BMI SDS	+0.8 (0.3-1.3)	27	-0.3 (-0.5-(-) 0.1)	132	0.000
Bone age advancement (months)^b^	23.0 (3.0-48.0)	23	4.0 (1.0-19.0)	64	0.000
Estradiol (pg/mL)^a^	30.0 (18.0-137.0)	13	18.4 (13.0-85.0)	77	0.288
Inhibin B (pg/mL)	44.2 (23.9-64.4)	12	30.2 (25.6-34.7)	50	0.165
TSH (IU/L)	2.6 (2.1-3.1)	17	2.5 (2.2-2.8)	73	0.827
SHBG (nmol/L)	81.1 (60.2-101.9)	12	114.5 (102.3-126.8)	37	0.007
LH0 (IU/L)^b^	0.4 (0.1-3.0)	19	0.3 (0.05-1.0)	46	0.016
LH30 (IU/L)^b^	7.2 (3.0-45.0)	19	3.8 (0.6-24.0)	47	0.000
FSH0 (IU/L)^b^	2.9 (0.9-8.4)	19	2.85 (0.6-17.3)	46	0.891
FSH30 (IU/L)^b^	9.3 (2.3-27.7)	19	18.9 (0.8-77.9)	47	0.001
Basal LH/FSH ratio^b^	0.2 (0.02-0.71)	19	0.1 (0.02-1.25)	46	0.031
Peak LH/FSH ratio^b^	0.7 (0.26-2.67)	19	0.2 (0.05-1.25)	47	0.000

Results are presented as mean values with 95 % confidence intervals

^a^Indicates non-parametric tests where results are presented as median values and ranges. ^b^Indicates parametric test with ln-tranformed data where results are presented as the untransformed median values and ranges

A greater percentage of girls with PP were of non-European origin (*p* = 0.012) and presented with a higher BMI SDS (*p* < 0.001) compared to the girls with PT.

85.2 % of the girls with PP and 38.4 % of the girls with PT had a bone age examination. Bone age was advanced in all of the PP girls with a median advance of 23 months and in the PT girls bone age deviated from chronological age with a median of plus 4 months (*p* < 0.001).

### Biochemical characteristics

SHBG levels were decreased in the PP group (*p* = 0.007), and there was no overlap between the PT and the PP group (Table 2). No between-group differences were observed for estradiol, inhibin B, and TSH (Table 2).

Nineteen girls (70.4 %) with PP, median age 6.0 years, and 47 girls (28.7 %) with PT, median age 2.3 years, underwent a GnRH test. Basal LH and stimulated values were significantly increased in the PP group compared with the PT group (*p* = 0.016 and *p* < 0.001, respectively), whereas the stimulated FSH value was decreased in the PP group compared with the PT group (*p* = 0.001) (Table 2). A stimulated LH-response ≥5 IU/l was recorded in 57.9 % of the girls with PP and in 38.3 % of the girls with PT.

**Fig. 3:**
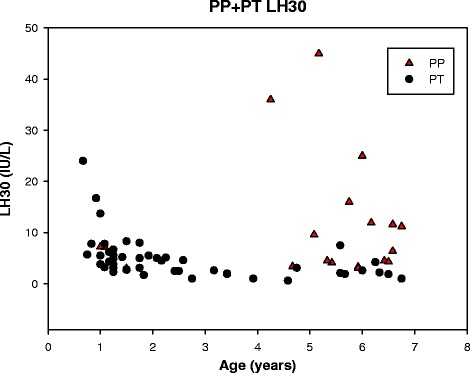
Gonadotropin releasing hormone stimulated luteinizing hormone (LH) in girls with precocious puberty (PP) and premature thelarche (PT) in the age groups ½-7 years

**Fig. 4:**
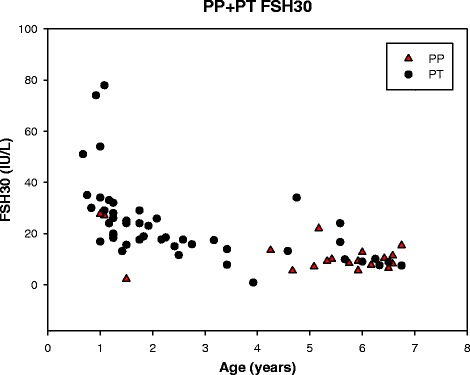
Gonadotropin releasing hormone stimulated follicle stimulating hormone (FSH) in girls with precocious puberty (PP) and premature thelarche (PT) in the age groups ½-7 years

**Fig. 5:**
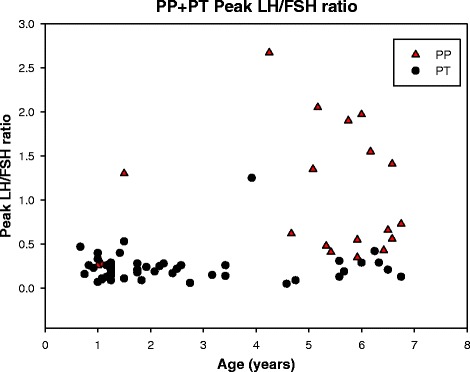
Gonadotropin releasing hormone stimulated LH/FSH ratio in the age groups ½-7 years

Stimulated LH/FSH ratio was 0.7 (0.3; 2.7) for the PP-group and 0.2 (0.05; 1.3) for the PT-group (*p* < 0.001), Table 2. The LH/FSH ratio was ≥1 for 8 (42.1 %) of the PP girls and 1 (2.1 %) of PT girls (due to a low stimulated FSH value). The basal LH/FSH ratio also indicated a significant difference between the groups (*p* = 0.03), Table 2. The stimulated LH and FSH concentrations are presented in Figs. 3 and 4, respectively. The youngest girls exhibited the largest stimulated FSH responses. Some girls with PT in the age group ½-3 years had a stimulated LH response comparable to the stimulated LH response in girls presenting PP 5–7 years old. The stimulated LH/FSH ratios was less than 0.6 in 46 of 47 girls with PT and higher than 0.6 in 11 of 19 girls with PP, Fig. 5.

## Discussion

Breast development before the age of 7 years of age is often an isolated and self-limiting condition. It can, however, also be the first sign of precocious puberty, which needs further examinations and medical treatment to prevent psychosocial, metabolic, and cardiovascular adverse events. While it is well known that the incidence of premature thelarche was increasing in the period from 1940 to 1984 [11] and that the timing of pubarche is still declining [3, 19, 20] it remains to be investigated, if the incidence of premature thelarche has continued to increase. We addressed this question and the novel finding of our study is that the incidence of girls referred because of premature thelarche before the age of 7 years is increasing. We excluded girls below 6 months of age because precocious puberty in this age group is extremely rare i.e. in a study of 302 girls with thelarche at birth or during the first 6 months of life, none of them developed precocious puberty [12]. Girls older than 7 years were also excluded because of the trend towards onset of pubertal development in this age group (”nonprogressive precocious puberty”) as a part of the normal puberty [21]. The continuous increase in girls referred for premature thelarche has also been observed in France as published at a major international congress on pediatric endocrinology [22]. The increasing number of referrals in our study indicates a higher incidence of PT and may have several explanations such as changes in the ethnic composition, increasing BMI and increased number of referrals for premature thelarche because of greater awareness of the condition in the general population and by the general practitioners.

Differentiating premature thelarche from early stages of precocious puberty in girls is difficult. Therefore, for improving future clinical care of girls with premature thelarche and precocious puberty, we constructed a database on clinical and biochemical data of the girls in our study cohort. Our aim was to identify signs, which can help differentiate girls with premature thelarche from girls with early stages of precocious puberty, but also to draw attention to the challenges in using conventional variables for PP diagnostics in distinguishing PT and PP in the age group ½-7 years.

The most interesting and clinical useful discovery in our study was, that we observed decreased SHBG levels in girls with precocious puberty and observed no overlap of SHBG concentrations in the two groups. Circulating levels of SHBG and androgens correlate inversely and SHBG usually plateau until puberty where after a decline is recorded [23]. We did not routinely measure androgens in our cohort but speculate that a puberty-associated increase of androgen levels caused a decrease of systemic SHBG in girls with precocious puberty. 25 % of the PP girls who underwent a SHBG analysis had pubic hair Tanner stage 2.

Several other parameters also differed between the groups: Chronological age, BMI SDS, ethnic origin, bone age advancement, basal and stimulated LH, stimulated FSH, basal and stimulated LH/FSH ratio.

Fourteen percent of the girls, who were referred because of breast development, progressed to precocious puberty, but the risk was strongly dependent on the age as only 3 % of girls between 6 months and 3 years, and 41 % girls older than 3 years of age developed precocious puberty. This supports the notion that breast development in the first few years of life is most often a physiological condition that stabilizes or regresses spontaneously [11, 14, 24–26] and why clinical follow-up without hormonal examinations may be the primary option in most cases in the youngest age group. Our data are in line with two earlier reports, where 13 and 14 % of girls with premature thelarche, respectively, developed precocious puberty [13, 14], but contrasts another study, that reported a larger proportion of girls e.g. 6.1 % out of 148 aged 6 months to 3 years progressed from premature thelarche to precocious puberty [12].

Non-European origin was associated with increased risk for precocious puberty in our study, which supports previous reports on the positive association between precocious puberty and ethnic origin [27, 28].

Advanced bone age is a hallmark of precocious puberty [9] and was quite increased in our group of girls with precocious puberty as compared with the girls with premature thelarche.

According to Bizzarri et al. [12] the combined measurement of basal LH and longitudinal diameter of the uterus represents a reliable screening approach to identify subjects who should undergo GnRH testing. Our data set revealed that only a small proportion of our study cohort had an ultrasonography examination and the data quality was low, and ultrasound data were therefore not included in our study.

The gold standard confirmatory laboratory test for central idiopathic precocious puberty for girls older than 6 years of age is a GnRH stimulated LH response in the pubertal range [29], but, so far, no reference interval exists for girls below 6 years of age. When comparing the group of girls progressing to precocious puberty with the group of girls with premature thelarche, we observed an increased basal and stimulated LH response and an increased basal and stimulated LH/FSH ratio. Neely et al. reported that basal LH concentrations were above 0.3 IU/L in all girls in late puberty [30]. This was indeed in line with 89.5 % of the girls in our cohort with precocious puberty, but 69.6 % of the girls with premature thelarche also exhibited increased LH concentrations leaving basal LH measurements inadequate to identify girls with premature thelarche. Bizzarri et al. reported that a basal LH concentration above 0.2 IU/L was the best positive and negative predictor of premature thelarche progressing into precocious puberty in 0 to 3 year old girls [12], but contrasts the basal LH concentrations in our study, where girls in that age group with premature thelarche presented with a median basal LH concentration of 0.3 IU/L. Only 57.9 % of our girls with precocious puberty had a stimulated LH above 5.0 IU/L, and girls with premature thelarche in age group ½-3 years exhibited a stimulated LH comparable to the stimulated LH in the precocious puberty group in 5–7 year old girls. A GnRH-stimulated LH/FSH ratio ≥1 has been considered to have high sensitivity and specificity for differentiating between precocious puberty and premature thelarche [31]. However, applying a stimulated LH/FSH ratio ≥1 as a cut-off value could not discriminate all our girls with precocious puberty from girls with premature thelarche, which was also observed in a recent study [12].

Surprisingly, we did not observe changes in estradiol, inhibin B, and TSH levels in girls with precocious puberty, and speculate that the lack of significance is attributed to a limited data set and a large inter-individual variability. E.g. the girl with the highest estradiol and increased LH response to the GnRH test, who was assigned to the premature thelarche group, underwent an extensive diagnostic program and an appropriate observation period and proved not to have precocious puberty, but rather premature thelarche, unsustained precocious puberty [32] or prolonged minipuberty. Usually minipuberty is considered to affect girls up 6 months of age but may last for a prolonged period [33].

The retrospective study design is a limitation and implies that data are not homogenous for the enrolled girls causing some missing information. The missing data on some of the girls, especially in the group with premature thelarche, also made comparison of the studied parameters difficult and indicates a selection bias for performing for example GnRH testing, bone age examination, and pelvic ultrasonography. Furthermore, only girls born before 2006 had a 7-year follow up, resulting in an underestimated incidence in the years 2006 to 2012. The assay for LH, FSH, SHBG and TSH changed from chemiluminescence immunoassay to electrochemiluminescence immunoassay during the study period, however reference values did not change for our age-group.

### Conclusion

We observed that the incidence of referral for premature thelarche is increasing in the Central Region of Denmark. It remains to be investigated if this earlier breast development over time will advance the entire sexual maturation. The incidence for premature thelarche in our study may serve as reference for future studies investigating secular trends of clinical signs of early pubertal development.

SHBG concentrations appeared to be useful to differentiate girl with premature thelarche from girls with precocious puberty. The sensitivity and specificity of SHBG in differentiating PT from PP needs to be further tested in future prospective studies. No other isolated clinical characteristic or hormonal parameter predicted the progression of premature thelarche to precocious puberty in girls below 7 years of age. Age, BMISDS, ethnicity, bone age, stimulated and basal LH/FSH ratio are all useful variables for differentiating PP from PT, but with considerable overlap between the PT and the PP group. It is notable that the stimulated LH value in 1–3 years old girls with PT may be as high as in 5–7 years old girls with PP. Therefore, in clinical practice, hormonal and x-ray testing in the younger girls should be limited to those with atypical or clearly progressive findings during follow-up. The GnRH test may, in future studies, also be useful to differentiate between premature thelarche and precocious puberty in girls below 6 years of age, but requires establishing of a reference interval in this age group.
